# Exploring the Cardiotoxicity Spectrum of Anti-Cancer Treatments: Definition, Classification, and Diagnostic Pathways

**DOI:** 10.3390/jcm12041612

**Published:** 2023-02-17

**Authors:** Ciro Mauro, Valentina Capone, Rosangela Cocchia, Filippo Cademartiri, Ferdinando Riccardi, Michele Arcopinto, Maie Alshahid, Kashif Anwar, Mariano Carafa, Andreina Carbone, Rossana Castaldo, Salvatore Chianese, Giulia Crisci, Roberta D’Assante, Mariarosaria De Luca, Monica Franzese, Domenico Galzerano, Vincenzo Maffei, Alberto Maria Marra, Alfredo Mazza, Brigida Ranieri, Anna D’Agostino, Salvatore Rega, Luigia Romano, Sarah Scagliarini, Chiara Sepe, Olga Vriz, Raffaele Izzo, Antonio Cittadini, Eduardo Bossone, Andrea Salzano

**Affiliations:** 1 Cardiology Division, Antonio Cardarelli Hospital, Via Cardarelli, 9, 80131 Naples, Italy; 2 Department of Advanced Biomedical Sciences, University of Naples Federico II, Via Sergio Pansini, 5, 80131 Naples, Italy; 3 Department of Radiology, Fondazione G. Monasterio CNR-Regione Toscana, Via Moruzzi, 1, 56124 Pisa, Italy; 4 Oncology Unit, Antonio Cardarelli Hospital, Via Cardarelli, 9, 80131 Naples, Italy; 5 Department of Translational Medical Sciences, Federico II University, 80138 Naples, Italy; 6 The Heart Centre, King Faisal Specialist Hospital & Research Centre, Riyadh 11564, Saudi Arabia; 7 Emergency Medicine Division, Antonio Cardarelli Hospital, Via Cardarelli, 9, 80131 Naples, Italy; 8 Unit of Cardiology, Department of Translational Medical Sciences, University of Campania “Luigi Vanvitelli”, Monaldi Hospital, 80131 Naples, Italy; 9 IRCCS SYNLAB SDN, Via Emanuele Gianturco, 113, 80143 Naples, Italy; 10 Post Operative Intensive Care Division, Antonio Cardarelli Hospital, 9, 80131 Naples, Italy; 11 Unit of Cardiology, Camerino Hospital, 62032 Macerata, Italy; 12 Department of Public Health, University Federico II of Naples, Via Sergio Pansini, 5, 80131 Naples, Italy; 13 Department of General and Emergency Radiology, Antonio Cardarelli Hospital, Via Cardarelli, 9, 80131 Naples, Italy; 14 Technical Nursing and Rehabilitation Service (SITR) Department, Cardarelli Hospital, 80131 Naples, Italy

**Keywords:** cardio-oncology, cancer therapy-related cardiovascular toxicity, chemotherapy

## Abstract

Early detection and treatment of cancer have led to a noticeable reduction in both mortality and morbidity. However, chemotherapy and radiotherapy could exert cardiovascular (CV) side effects, impacting survival and quality of life, independent of the oncologic prognosis. In this regard, a high clinical index of suspicion is required by the multidisciplinary care team in order to trigger specific laboratory tests (namely natriuretic peptides and high-sensitivity cardiac troponin) and appropriate imaging techniques (transthoracic echocardiography along with cardiac magnetic resonance, cardiac computed tomography, and nuclear testing (if clinically indicated)), leading to timely diagnosis. In the near future, we do expect a more tailored approach to patient care within the respective community along with the widespread implementation of digital health tools.

## 1. Introduction

Early detection and treatment of cancer have led to a noticeable reduction in both morbidity and mortality of malignancies [1]. However, conventional chemotherapeutics and some of the newer anti-cancer signaling inhibitors (e.g., antibodies and protein kinase inhibitors or immune checkpoint inhibitors (ICI)) could exert cardiovascular (CV) side effects impacting the patient’s survival and quality of life, independent of the oncologic prognosis [2,3].

**Figure 1:**
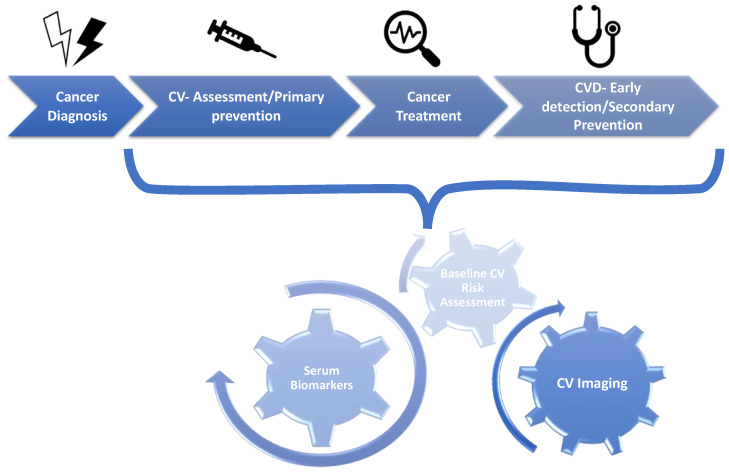
Cardio-oncology: an integrated approach throughout cancer disease [7]. Abbreviations: CV, cardiovascular, CVD, cardiovascular disease.

**Figure 2:**
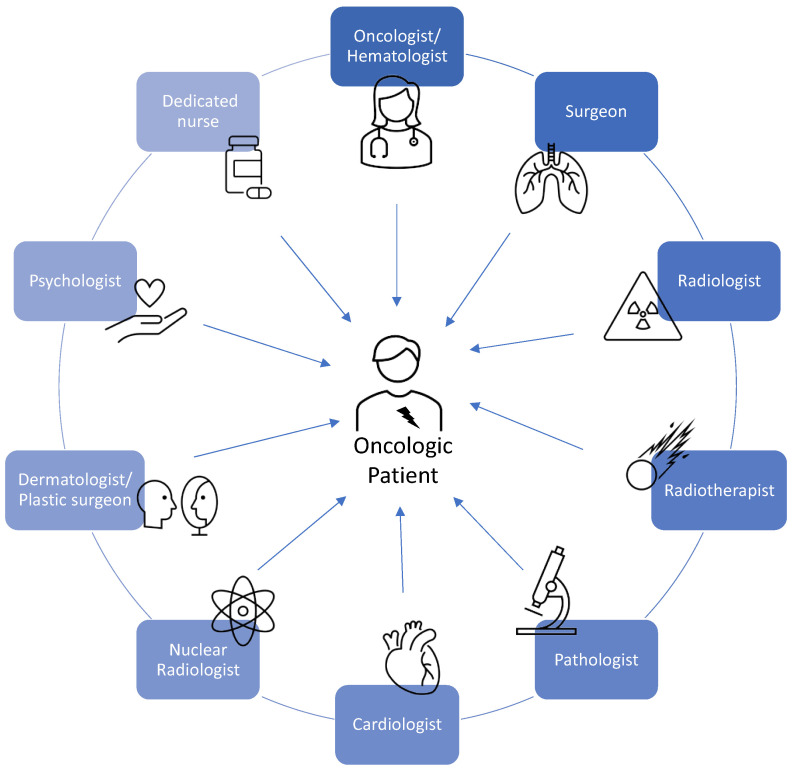
An integrated multidisciplinary approach in management of cancer patients [7].

In this regard, several scientific associations of cardiologists and oncologists have made an effort to better define the management of adverse sequelae of cancer therapy-related cardiovascular toxicity (CTR-CVT), leading to a new discipline known as cardio-oncology [4,5,6] (Figure 1 and Figure 2). 

Therefore, the aim of this paper is to discuss the full spectrum of CTR-CVT and related diagnosis through a pragmatic approach mainly based on pre-existing guidelines. 

## 2. The Spectrum of Cardiotoxicity

According to current 2022 European Society of Cardiology (ESC) guidelines on cardio-oncology [7], CTR-CVT includes a wide spectrum of CV side effects (Table 1) leading to CV disease (CVD): (i). Cancer therapy-related cardiac dysfunction (CTRCD); (ii). Myocarditis; (iii). The broad spectrum of vascular toxicities, both symptomatic and asymptomatic; (iv). Arterial hypertension; (v). Cardiac arrhythmias. Furthermore, pericardial and valvular heart disease (VHD) and pulmonary hypertension (PH) should also be considered [7]. 

### 2.1. Cancer Therapy Related Cardiac Dysfunction

CTRCD is classified into two main clinical categories: (i) Asymptomatic CTRCD (mild: left ventricular ejection fraction (LVEF) ≥50% accompanied by a relative decline in global longitudinal strain (GLS) of >15% from baseline and/or a new increase in cardiac biomarkers; moderate: new LVEF reduction ≥10% to an LVEF of 40–49% or new LVEF reduction <10% to an LVEF of 40–49% and an either new relative decline in GLS of >15% from baseline or a new increase in cardiac biomarkers; severe: new LVEF reduction to <40%); (ii) Symptomatic CTRCD (overt heart failure (HF), ranging from mild to very severe)) [7]. Of note, the InterTAK Registry investigators have reported a higher rate of Takotsubo syndrome (TTS) in cancer patients compared with the general population as a consequence of cancer treatments toxicity (e.g., 5-fluorouracil (5-FU), ICI, vascular endothelial growth factor inhibitors (VEGFi) and tyrosine kinase inhibitors (TKIs)) along with increased emotional stress and elevated sympathetic tone [8].

#### Specific CTRCD

- Anthracyclines (e.g., doxorubicin, epirubicine, daunorubicin) are frequently employed treatment of solid tumors and hematological tumors but could contribute to the development of left ventricular dysfunction (LVD) (from myocardial cell injury to impaired LVEF and symptomatic HF) [9]. The proposed mechanisms of anthracycline-related cardiomyopathy include the transport of anthracyclines across the cardiomyocyte cell membrane, generation of reactive oxygen species (ROS) through the inhibition of topoisomerase 2β (resulting in activation of cell death pathways and mitochondrial dysfunction), generation of cardiotoxic anthracycline metabolites, and sarcomere disruption [2,10].
- Alkylating agents, such as cyclophosphamide, given in high doses before bone marrow transplantation, may cause HF due to several pathological effects as direct endothelial injury followed by extravasation of toxic metabolites that damage myocytes, interstitial hemorrhage, and edema. Furthermore, an ischemic myocardial injury could be the result of intracapillary microemboli. Cyclophosphamide may also damage the inner mitochondrial membrane of cardiomyocytes, most likely through the induction of oxidative stress [11].
- Cardiotoxicity of cisplatin, used for solid cancers (e.g., testicular, lung, cervical, and ovarian cancers), may result either from direct toxic action on cardiac myocytes or from ROS production, followed by the induction of oxidative stress and the switch to a prothrombotic condition [12]. Of note, platinum-based drugs need an infusion of high intravenous volumes to avoid cardio-toxicity [2,11].
- Immunotherapies and targeted therapies—implying inhibition of human epidermal growth factor receptor 2 (HER2) signaling with either antibodies (trastuzumab, pertuzumab) or TKIs (lapatinib)—have ameliorated survival of patients with HER2-positive breast cancer [2,13]. In addition, 3% to 7% of patients who receive trastuzumab monotherapy develop cardiac dysfunction (from asymptomatic LVEF decline to HF), which is usually reversible with drug interruption and/or HF treatment [2]. This percentage is even higher when trastuzumab is administrated after anthracyclines treatment [2].
- Both antibodies and protein kinase vascular endothelial growth factor (VEGF) signaling pathway inhibitors, used in several solid cancers (e.g., colorectal and lung cancer), induce LVD and HF mainly due to cardiac hypertrophy and mitochondrial abnormalities [2]. In particular, sorafenib-mediated inhibition of RAF1 and BRAF kinase activity will disrupt signaling through the extracellular signal-regulated kinase (ERK) kinase cascade, which is believed to have a role in heart cell survival, especially under conditions of stress [2].
- HF due to TKIs of BCR-ABL (e.g., imatinib, employed in chronic leukemia) has not been uniquely confirmed. However, these drugs could lead to significant mitochondrial dysfunction with loss of membrane potential, the release of cytochrome c, and markedly impaired energy generation with a significant decline in adenosine triphosphate (ATP) concentration, which is crucial to cardiomyocyte contractile function [2].
- Antimicrotubule agents (taxanes, such as docetaxel), frequently used in breast cancer, may be safer than anthracyclines in patients with pre-existing LVD. However, asymptomatic decrease in LVEF as well as overt congestive HF have been observed in patients previously treated with anthracycline and when docetaxel was combined with trastuzumab treatment for HER2-positive disease [2,11,14].
- Several investigators have demonstrated LVD related to proteasome inhibitors (PI), used in multiple myeloma, as a direct consequence of the inability of proteasomes to degrade dysfunctional or unneeded proteins in cardiomyocytes [2].
- ICIs may cause (through not entirely known mechanisms of action) myocarditis as well as non-inflammatory HF syndromes including Takotsubo syndrome [7].
- Chimeric antigen receptor T cell (CAR-T) therapies can be associated with clinically silent elevation in cardiac troponins (cTn) to decompensated HF [7,15].
- In addition to chemotherapy, radiation-induced CVD (increased risk for systolic and diastolic (more likely) heart failure) may be observed [16]. The related pathophysiological mechanisms are complex, including deoxyribonucleic acid damage, oxidative stress, and the release of inflammatory and profibrotic cytokines, leading to vascular and myocardial fibrosis, and as a result, the development of stenosis in radiated coronary arteries, subclavian, and carotids [2,16].

### 2.2. Myocarditis

Myocarditis is usually associated with ICI, which has recently been implemented to treat resistant malignancies [17,18]. The diagnosis of ICIs myocarditis includes cTn elevation along with diagnostic cardiac magnetic resonance (CMR)-specific markers based on updated Lake Louise criteria after exclusion of acute coronary syndromes (ACS) and (although rare) acute infectious etiology [7]. Endomyocardial biopsy is indicated in the case of an ongoing unstable hemodynamic state or uncertain diagnosis [19].

### 2.3. Vascular Toxicity

Vascular chemotherapeutic agents’ toxicities include a broad spectrum of cardiovascular manifestations, both asymptomatic (atherosclerosis and abnormal vasoreactivity) and symptomatic (stroke/transient ischemic attack, myocardial infarction, ACS and chronic coronary syndromes, peripheral artery disease (PAD), vasospastic and microvascular angina, and Raynaud’s phenomenon).

#### 2.3.1. Coronary Artery Disease (CAD)

Mechanisms of coronary artery toxicity consist (and may coexist) in direct vasospastic effect (fluoropyrimidines), endothelial injury (fluoropyrimidines, Veggie, radiotherapy), acute arterial thrombosis (cisplatin), vasculitis (ICI), and long-term changes of lipid metabolism with resulting early arteriosclerosis (ALK inhibitors, BCR-ABL TKIs) [2]. There is also evidence that previous radiotherapy of mediastinum may cause or accelerate drug-related coronary disease as it provokes endothelial injury and plaque rupture, and favors thrombosis with a risk proportional to irradiation dose [2,16].

#### 2.3.2. Peripheral Vascular Disease and Stroke

Severe atherosclerotic and non-atherosclerotic PAD in the lower extremities can occur in patients (up to 30%) treated with nilotinib, ponatinib, or BCR-ABL TKIs (used for chronic myeloid leukemia). TKIs can induce a vasospasm on stenosis in arteries and exert proatherogenic effects on endothelial cells [20]. Bleomycin, cyclophosphamide, vinka alcaloids, cisplatin, methotrexate, 5-FU, and paclitaxel could cause peripheral arterial toxicities such as Raynoud’s phenomenon and ischemic stroke [21].

After mediastinal, cervical, or cranial radiotherapy, the risk of stroke is doubled [22]. The proposed mechanisms, on one hand, are endothelial damage and thrombus formation in small vessels and, on the other hand, vasa vasorum occlusions, necrosis/fibrosis, and accelerated atherosclerosis of the medium or large vessels (including carotid, aorta, subclavian, and iliofemoral) [2]. 

#### 2.3.3. Thromboembolic Disease

In addition to cancer itself and the patient’s risk profile, venous thromboembolism (VTE) can be directly related to chemotherapy and its administration route (use of indwelling venous catheters) [23]. In this regard, it should be noted that VTE is among the most frequent causes of death after cancer surgery [2]. Recommendations for both prophylaxis and treatment of VTE in patients with cancer and COVID-19 are similar to those of patients without COVID-19 [24,25]. As a note, intra-arterial thrombotic events may also but rarely occur in cancer patients under anthracyclines, taxanes, and platinum-based chemotherapies [2,26].

### 2.4. Systemic Arterial Hypertension (HTN)

VEGFi may induce HTN through several pathophysiological mechanisms (i.e., nitric oxide pathway inhibition, vascular rarefaction, renal thrombotic microangiopathy, etc.) [27,28,29]. 

Other chemotherapeutics (BCR-ABL TKI, brigatinib, ibrutinib, fluoropyrimidines, cisplatin, enzalutamide) could also provoke HTN, which is also often fostered by the use of corticosteroids and non-steroidal anti-inflammatory drugs along with factors as stress and pain [7].

### 2.5. Arrhythmias

Both tachyarrhythmia and bradyarrhythmia may be related to cancer-related therapy [2,30]. Atrial fibrillation (AF) is the most frequent chemotherapy (alkylating agents, anthracyclines, antimetabolites, etc.) and/or radiotherapy-related supraventricular tachyarrhythmia. [31]. It may also be commonly observed after cancer surgery. However, most cancer patients present several AF predisposing factors (advanced age, electrolyte abnormalities, hypoxia, metabolic disorders, etc.) that need to be considered by the treating multidisciplinary team during the clinical evaluation. 

QT prolongation (most frequently caused by arsenic trioxide) and ventricular arrhythmias are usually related to several chemotherapeutics (alkylating agents, amsacrine, antimetabolites, arsenic trioxide, doxorubicin, etc.) as well as radiotherapy should also be taken into account when treating cancer patients [2].

Less frequently, chemotherapy can cause sinus node dysfunction leading to bradycardia, and heart blocks [2,32]. As a note, conduction defects may also appear after many years after neck radiotherapy directly linked to autonomic dysfunction secondary to injury and fibrosis of the carotid sinus [16].

### 2.6. Pericardial Disease

Acute pericarditis (with/without large effusion leading to hemodynamic instability such as tamponade) may be observed with the use of several chemotherapeutic drugs (predominantly anthracyclines, cyclophosphamide, cytarabine, bleomycin, ICI) and/or after high dose radiotherapy as in the case of mediastinal tumors [2,33,34]. Furthermore, chronic constrictive pericarditis may rarely occur several years later in high-dose radiotherapy [2,16,35].

### 2.7. Valvular Heart Disease 

Chemotherapeutic agents do not directly target cardiac valves, but in cancer patients VHD may be detected as secondary to ventricular dysfunction (mitral and/or tricuspid regurgitation) and/or endocarditis (bacteremia and sepsis due to chemotherapy-associated pancytopenia and/or indwelling catheters) [4,36,37,38]. Radiation-induced VHD has been also reported among ~10% of treated patients [2,39]. It should be noted that left-sided valves are more commonly affected, with the aortic valve being the most involved one [35,40].

Cancer therapy related-VHD is usually characterized by an unusual pattern of calcification extending from the base of the anterior mitral leaflet to the noncoronary aortic sinus [41]. Calcification typically spares the tips of mitral valve leaflets and does not lead to commissural fusion [16]. 

### 2.8. Pulmonary Hypertension

PH is a rare but serious complication of anti-cancer treatments and stem cell bone marrow transplantation [7,42]. In this regard, dasatinib (used for chronic myelogenous leukemia) can generate serious precapillary PH (through smooth muscle cell proliferation in pulmonary arterioles and vasoconstriction (group 1)) that is usually reversible after drug cessation and consequent relative change with another TKI, such as nilotinib [43]. Furthermore, severe pulmonary veno-occlusive disease (group 1) has been reported among cancer patients treated with cyclophosphamide and other alkylating agents. PH may be also related to CTRCD (group 2), pulmonary fibrosis (secondary to thoracic radiation or bleomycin) (group 3), central venous catheter and/or malignant tumors (e.g., renal carcinoma) (group 4), or multifactorial mechanisms (group 5) [7].

## 3. Diagnostic Pathways

Cardiotoxicity diagnosis relies on timely high clinical suspicion (anamnesis, physical examination, and electrocardiogram) triggering specific serum biomarkers (namely natriuretic peptides (NPs) and cTn) and appropriate imaging techniques (transthoracic echocardiography (TTE) along with CMR, cardiac computed tomography (CCT) and nuclear testing (if clinically indicated)). 

### 3.1. Clinical Assessment

A careful baseline clinical assessment (anamnesis, physical examination, and electrocardiogram) is recommended in all patients starting cancer treatment to estimate the personal risk of developing CTR-CVT. According to current 2022 ESC guidelines on cardio-oncology [7], the baseline risk assessment should be easily pursued through drug related-proformas provided by the Heart Failure Association—International Cardio-Oncology Society (HFA-ICOS) for all patients who are expected to receive potentially cardiotoxic cancer therapy [7]. In this regard, cancer patients are divided into four CTR-CVT baseline risk categories (low, moderate, high, and very high) and in turn, referred to the specific related surveillance program [7].

### 3.2. Serum Biomarkers

Amongst serum biomarkers validated in the detection of cardiotoxicity, NPs and cTn are the most used in daily clinical practice [44,45].

NPs, including B-type natriuretic peptide (BNP) and N-terminal pro-B-type natriuretic peptide (NT-proBNP), are quantitative and qualitative markers for the presence and severity of hemodynamic cardiac stress as in HF [46]. 

Short-term temporal changes in high-sensitivity cardiac troponin (hs-cTn) concentrations can differentiate acute disease (rapid rise and/or fall) from chronic cardiomyocyte injury (persistent slight elevation) [47]. As noted, chronic cTn elevation can be associated with the presence of comorbidities such as chronic kidney disease, diabetes mellitus, significant left ventricular hypertrophy, and HF [48].

According to Lyon et al. and based on our experience, serial NPs and hs-cTn measurements should be undertaken in all patients with cancer at risk of CTRCD [7,44]. 

Several novel biomarkers (e.g., myeloperoxidase, high-sensitivity C-reactive protein, sFlt-1, placental growth factor, growth differentiation factor-15, galectin-3, arginine, heart-type of fatty acid binding protein, glycogen phosphorylase BB) have been studied to detect earlier or subclinical cardiotoxicity and institute cardioprotective strategies beyond the prediction currently offered by cTn and NPs [49,50]. In addition, markers of the immune system, (i.e., immunoglobulin E), could help to identify patients at increased risk for doxorubicin and trastuzumab cardiotoxicity [51]. Lastly, recent studies in breast cancer patients have indicated microRNAs as a potential biomarker in the detection of cancer drug-induced cardiotoxicity [52], as shown in (Table 2) [51,53,54,55,56,57,58,59,60].

### 3.3. Cardiovascular Imaging

#### 3.3.1. Transthoracic Echocardiography

**Figure 3:**
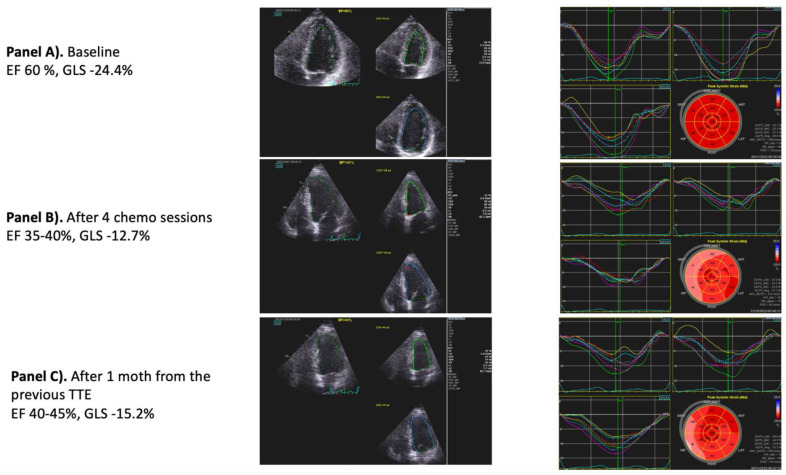
Echocardiography in the evaluation of chemotherapy CV side-effects. A 58-year-old female patient with known dyslipidemia and no other cardiovascular disease (**Panel A**). The patient was affected by stage IIIC high-grade serous ovarian cancer. She underwent four sessions of taxol/carboplatin protocol (S/P 4 cycles) before surgical debulking. After the last chemo session, she developed shortness of breath during mild physical effort which she never experienced before. At TTE, a significant reduction in LVEF and GLS compared with the baseline was seen (**Panel B**). The patient was put on metoprolol and Lisinopril. Atorvastatin and TTE were repeated after 1 month with evidence of mild improvement in LVEF and GLS (**Panel C**). Abbreviations: EF, left ventricular ejection function; GLS, global longitudinal strain; TEE, transthoracic echocardiography.

TTE (non-invasive, radiation-free, and virtually implementable in any clinical scenario) is recommended as the first-line imaging modality in cancer patients (Figure 3).

In addition, it provides accurate information regarding heart structure, function, and derived Doppler intracardiac hemodynamics (Table 3). 

It should be emphasized that advanced ultrasound techniques (namely strain and 3-dimensional echocardiography) may provide new insights into the evaluation of chemotherapy and/or radiotherapy CV side effects. In this regard, GLS has emerged as a new marker of early subclinical ventricular dysfunction not usually detected by conventional two-dimensional parameters such as LVEF [62]. Therefore, if available, a strain imaging technique is recommended every time an echocardiographic exam is performed [7]. 

#### 3.3.2. Transephofageal Echocardiography (TEE)

TEE, performed by TEE-experienced physicians, gives key information concerning the diagnosis of infective endocarditis, acute aortic syndromes, intracardiac shunts, cardiac (suspected tumor or thrombus), and pericardial masses [63]. Absolute contraindications include esophageal diseases, recent gastroesophageal surgery, and severe respiratory depression. Informed patient consent should be obtained [64]. 

#### 3.3.3. Vascular Ultrasound

In the suspicion of cancer therapy-induced PAD, a repeatable and feasible non-invasive method such as duplex ultrasound (DUS) is a first step in the vascular workup both for screening and diagnosis. It includes B-mode echography, pulsed-wave, continuous, color, and power Doppler modalities to detect vascular lesions and quantify their severity through velocity criteria [65]. DUS should be considered also considered for the detection of abdominal aorta aneurysms [66]. In addition, computed tomography angiography (CTA) and/or magnetic resonance angiography (MRA) are indicated for further anatomical characterization of vascular lesions and guidance for optimal revascularization strategy [65].

#### 3.3.4. Cardiac Magnetic Resonance

When TTE is unavailable or non-diagnostic, as in cachectic patients or in patients who have previously undergone left breast or left chest surgery and/or radiotherapy [61], or in case of patients with complex pre-existing heart diseases [61] such as hypertrophic cardiomyopathy, using alternative imaging modalities such as CMR should be considered for serial monitoring of LV size and function.

Although less feasible and more expensive, CMR (a radiation-free technique) has greatly improved accuracy and reproducibility in the estimation of cardiac function structure and function [67,68]. CMR also gives useful data about the existence of previous myocardial infarction scar, diffuse fibrosis, and intracellular or interstitial edema during cancer treatment, often revealing the pathogenesis of cardiotoxicity from the different cancer drug classes and radiation [69,70]. Moreover, new evidence suggests that novel CMR indices may be more sensitive than other imaging modalities regarding anthracycline-induced damage [71]. In addition, CMR is particularly important when ICI-mediated myocarditis is suspected [72]. Lastly, it is an excellent test for the evaluation of pericardial diseases, cardiac masses, infiltrative (amyloidosis), and storage diseases [73,74]. Of note, CMR protocols for CTRCD evaluation differ in individual cases [75,76].

#### 3.3.5. Cardiac Nuclear Imaging

The historical method of planar imaging multigated acquisition (MUGA) scan is not recommended as a first-line surveillance cardiac imaging modality for cancer patients due to the possibility of using other radiation and accurate techniques, such as cardiac ultrasound and CMR modalities [7,77]. However, fluorodeoxyglucose positron emission tomography (18F-FDG PET) associated with computed tomography or magnetic resonance imaging may be useful to monitor cancer progression and, at the same time, potential drug-induced cardiotoxicity (i.e., ICI-mediated myocarditis when CMR is not available or contraindicated) [61]. 

#### 3.3.6. Coronary Computed Tomography Angiography and Imaging Stress Tests

Cardiac computed tomography (CCT) plays a major role in any situation in which there is a suspicion of significant CAD or even more suspected obstructive CAD; this would be the more restricted application of CCT, which is coronary computed tomography angiography (CCTA). The clinical diagnostic role of CCT, however, is not limited to that very simple task. CCT can deliver information that is at least as robust as CMR for the anatomy and function of the left/right ventricle, left/right atrium, valves, and pericardium. CCTA has been proposed to detect subclinical CAD as radiation-related coronary damage [78]. In radiotherapy survivors, the accuracy of CCTA and calcium score in the diagnosis of significant CAD is high and demonstrates excellent negative predictive value [79,80]. Notwithstanding, the timing of CCTA for surveillance in asymptomatic cancer survivors following high-dose radiation to the chest is unknown and requires further studies [61]. 

In patients with suspected angina, stress TTE/CMR (physical or pharmacological depending on the patient’s clinical status) is recommended to diagnose the presence and extent of myocardial ischemia and assess the need for therapeutic interventions [61,81].

**Figure 4:**
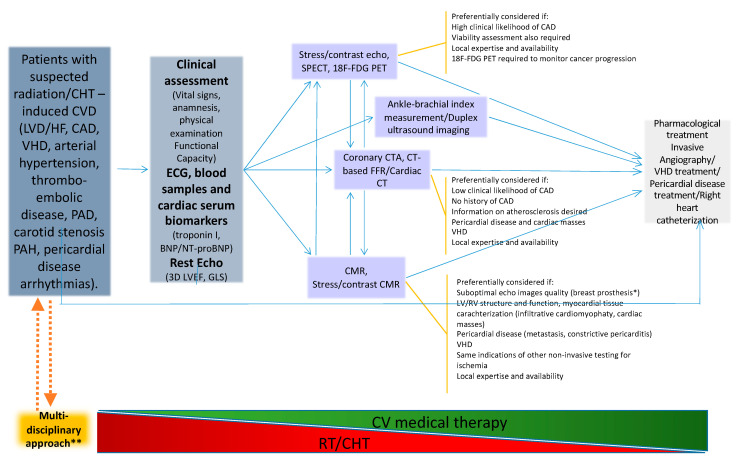
Management of cancer therapy-related CVD: a stepwise approach [2,7,36,38,82,83,84,85,86,87]. BNP = brain natriuretic peptide; CHT = chemotherapy; CMR = cardiac magnetic resonance; CT = computed tomography; CTA = computed tomography angiography; FFR = fractional flow reserve; CVD = cardiovascular disease; HF = heart failure; GLS = global longitudinal strain; LVD = left ventricular dysfunction; LVEF = left ventricular ejection function; NT-proBNP = N-terminal pro-brain natriuretic peptide; PH = pulmonary hypertension; RT = radiotherapy; VHD = valvular heart disease. * It is important to inquire about the presence of ferromagnetic components if the patient has breast tissue expanders. ** Including cardiologists, oncologists, radiotherapist oncologists, and hematologists.

A pragmatic multidisciplinary stepwise approach to cardiotoxicity is depicted in Figure 4.

A list of current guidelines on both cardio-oncology and specific CTR-CVT, used in preparing this review, is displayed in Table 4. 

## 4. Conclusions

CVR-CVT may have a strong impact on patients’ survival and quality of life, independent of the oncologic prognosis. 

In this regard, the combination of serum and imaging markers along with clinical assessment may improve early detection and treatment of cardiotoxicity. It is therefore pivotal to develop integrated multidisciplinary teams in order to provide the most appropriate management for the oncologic patient. In the near future, we do expect a more tailored approach to patient care along with the widespread implementation of digital health tools. 

## Figures and Tables

**Table 1:** Chemotherapy-related cardiovascular toxicity [7].

Chemotherapy	CTRCD	Myocarditis	Vascular Toxicities					Arterial Hypertension	Cardiac Arrhythmias	Pericardial Diseases	PH
			Stroke, TIA	MI, ACS, CCS, ATS	PAD	Vasospastic/ Microvascular Angina, Abnormal Vasoreactivity, Raynaud’s Phenomenon	VTE, Arterial Thrombosis				
Anthracycline	++										
HER2-targeted therapies	++										
Fluoropyrimidines	++ *	+	+	++		+		++	+ ^§^		
VEGFi	+			+		+	+	++	+ ^§^		
1st generation BCR-ABL TKI (Imatinib)	+							+			
2nd generation BCR-ABL TKI (Nilotinib, Dasatinib, Bosutinib)	+		+	+	+			+	+ ^§#^	+	+
3rd generation BCR-ABL TKI (Ponatinib)	+		+	+	+			++	+ ^#^	+	+
Alkylating agents (Cyclophosphamide, Melphalan)	+								++ ^#^		
Immunomodulatory drugs (Lenalidomide, Pomalidomide, Thalidomide)	+			+			++	+	+ ^#^		
Proteasome inhibitors (Bortezomib, Carfilzomib)	+						+	++	+ ^#^		+
Monoclonal antibodies (Daratumumab, Elotuzumab, Isatuxmab)	+						+	++	+ ^#^		
RAF inhibitors	+						++	++	+ ^§^		
MEK inhibitors	++							++			
Androgen deprivation therapy	+			+				++	+ ^#^		
ALK inhibitors								++	+ ^§^		
EGFR inhibitors	+						+				
CAR-T therapy	+								+ ^#^	+	
TIL-therapy			+	+	+	+					
ICI	+	++									

++ very common drug-related vascular toxicity. + common drug-related vascular toxicity. * CTRCD mainly in the form of TTS. ^§^ QT interval prolongation-related arrhythmias. ^#^ Atrial fibrillation. Abbreviations: ACS, acute coronary syndromes; ATS, atherosclerosis, CAR-T, Chimeric antigen receptor T cell; CCS, chronic coronary syndromes; CTRCD, cancer therapy-related cardiac dysfunction; EGFR, epidermal growth factor receptor; HER2, human epidermal growth factor receptor 2; ICI, immune checkpoint inhibitors; MI, myocardial infarction; PAD, peripheral artery disease; PH, pulmonary hypertension; TIA, transient ischemic attack; TKI, small molecule tyrosine kinase inhibitor; TIL, tumor-infiltrating lymphocyte; TTS, Takotsubo syndrome, VEGFi, vascular endothelial growth factor inhibitors; VTE, venous thrombosis and venous thromboembolism.

**Table 2:** Comparison of main characteristics of biomarkers potentially employed in cardiotoxicity detection.

Biomarkers	Disease	Characteristics
cTn	ACS, HF, PE	Cardiac-specific structural proteins composing the contractile apparatus of cardiomyocytes
BNP and NT-proBNP	HF, PE	Inactive form (prohormone) of BNP secreted by cardiomyocytes from increased transmural tension and neurohormonal stimulation (notably by noradrenaline and angiotensin II)
Myeloperoxidase	ACS	Released into extracellular fluid in response to inflammatory processes
High-sensitivity C-reactive protein	Aortic dissection, ACS	Marker of evolution of false lumen thrombosis
sFlt-1	Atherosclerotic cardiovascular disease	Markers of inflammation, endothelial function, and myocardial stress or injury.
Placental growth factor	ACS	Mitogen for endothelial cells; it can also act as a proinflammatory cytokine
Growth differentiation factor-15	Myocarditis	Marker of extracellular matrix degradation
Galectin-3	HF	Marker of cardiac and vascular fibrosis
Arginine	HTN, PH, atherosclerosis, and vasospasm	Endothelial dysfunction as Arg is the main source for the generation of NO via NOS.
H-FABP	ACS, HF, arrhythmia, PE	A dominant isoform present in the heart and skeletal muscles acting as marker of ongoing myocardial damage
Glycogen phosphorylase BB	ACS	Provide the fuel for the energy supply required for myocardial contraction
Immunoglobulin E	Cardiac dysfunction, HF	Dysregulation of the inflammatory response due could worse cardiac remodeling to cardiac injury
microRNAs	ACS	Non-coding RNAs which inhibit mRNA translation or induce its degradation; involved in all cardiac functions, including the conductance of electrical signals, heart muscle contraction, and growth

Abbreviations: ACS; acute coronary syndrome; BNP, B-type natriuretic peptide; cTn, cardiac troponins, cTn; HF, heart failure; H-FABP, heart-type of fatty acid binding protein; HTN, systemic arterial hypertension; NT-proBNP, N-terminal pro-B-type natriuretic peptide; PE, pulmonary embolism; PH, pulmonary hypertension; sFlt-1, soluble Flt-1, TTS, Takotsubo syndrome.

**Table 3:** Echocardiography protocol for cardio-oncology surveillance [33,38,61].

Parameters	Clinically Significant Changes
LV size and function	
LVEF by Simpson’s 2D, or (semi)automatic 3D	Drop >10% (percentage points) for 2D, >5% for 3D from pre-treatment value
2D/3D GLS, GCS	Relative reduction by >10–15% from pre-treatment value and to below lower limit of normal
LV 2D/3D systolic and diastolic volumes	Increase by 15 mL for ESV, 30–35 mL for EDV
RV function, pulmonary artery pressure, and volemia	
Markers of systolic RV function	TAPSE < 1.7 cm, FAC < 35%, RV free wall strain < 20%, 3D RVEF < 45%
Velocity of TR	Peak systolic TR velocity > 2.8 m/s
IVC diameter, collapse on inspiration	Dilatation > 2.1 cm or narrowing < 1.3 cm
VHD	Valvular calcification; valve regurgitation/stenosis
Pericardium	Pericardial effusion, cardiac tamponade, constrictive physiology

Abbreviations: 2D, two-dimensional; 3D, three-dimensional; EDV, end-diastolic volume; ESV, end-systolic volume; FAC, fractional area change; GCS, global circumferential strain; GLS, global longitudinal strain; IVC, inferior vena cava; LV, left ventricular; LVEF, left ventricular ejection fraction; RV, right ventricular; RVEF, right ventricular ejection fraction; TAPSE, tricuspid annular plane systolic excursion; TR, tricuspid regurgitation, VHD, valvular heart diseases.

**Table 4:** Employed guidelines on cardio-oncology and on specific CTR-CVT.

Topic	Guidelines	Ref.
Cardio-oncology	2022 ESC Guidelines on cardio-oncology. *Eur. Heart J.*	[5,7]
	Canadian Cardiovascular Society Guidelines for Cardiovascular Complications of Cancer Therapy. *Can. J. Cardiol.*	
HF	2021 ESC Guidelines for the diagnosis and treatment of acute and chronic heart failure. *Eur. Heart J.*	[36]
Infective endocarditis	2015 ESC Guidelines for the management of infective endocarditis. *Eur. Heart J.*	[37]
VHD	2021 ESC/EACTS Guidelines for the management of valvular heart disease. *Eur. Heart J.*	[38]
PH	2022 ESC/ERS Guidelines for the diagnosis and treatment of PH. *Eur. Heart J.*	[42]
PAD	2017 ESC Guidelines on the Diagnosis and Treatment of PAD. *Eur. Heart J*.	[65]
Aortic Disease	2022 ACC/AHA Guideline for the Diagnosis and Management of Aortic Disease. *Circulation*.	[66]
CCS	2019 ESC Guidelines for the diagnosis and management of CCS. *Eur. Heart J.*	[82]
ACS	2017 ESC Guidelines for the management of acute myocardial infarction in patients presenting with ST-segment elevation. *Eur. Heart J*.	[83,87]
	2020 ESC Guidelines for the management of acute coronary syndromes in patients presenting without persistent ST-segment elevation. *Eur. Heart J*.	
Pericardial diseases	2015 ESC Guidelines for the diagnosis and management of pericardial diseases. *Eur. Heart J*.	[84]
HTN	2018 ESC/ESH Guidelines for the management of arterial hypertension. *Eur. Heart J.*	[85]
AF	2020 ESC Guidelines for the diagnosis and management of AF. *Eur. Heart J*.	[86]

Abbreviations, ACC, American College of Cardiology; ACS; acute coronary syndrome; AF, atrial fibrillation; AHA, American Heart Association; CCS, chronic coronary syndromes; HF, heart failure; HTN, systemic arterial hypertension; PAD, peripheral arterial diseases; PH, pulmonary hypertension; VHD, valvular heart disease.

## Data Availability

The data are available from the corresponding author on reasonable request.

## Abbreviations

2D	two-dimensional
3D	three-dimensional
5-FU	5-fluorouracil
18F-FDG PET	Fluorine-18-Fluorodeoxyglucose Positron Emission Tomography
ACS	acute coronary syndromes
AF	atrial fibrillation
ATP	adenosine triphosphate
BNP	B-type natriuretic peptide
CAD	coronary artery disease
CAR-T	Chimeric antigen receptor T cell
CCS	chronic coronary syndromes
CCT	cardiac computed tomography
CCTA	Coronary computed tomography angiography
CMR	cardiac magnetic resonance
CTA	computed tomography angiography
cTn	cardiac troponins
CTRCD	cancer therapy related cardiac dysfunction
CTR-CTV	cancer therapy-related cardiovascular toxicity
CV	cardiovascular
CVD	cardiovascular diseases
DUS	duplex ultrasound
EDV	end-diastolic volume
EGFR	Epidermal Growth Factor Receptor
ESV	end-systolic volume
ERK	extracellular signal-regulated kinase
FAC	fractional area change
GCS	global circumferential strain
GLS	global longitudinal strain
HER2	human epidermal growth factor receptor 2
HER2i	HER2 inhibitors
HF	heart failure
hs-cTn	high sensitivity cardiac troponin
hs-cTnI	high sensitivity cardiac troponin I
hs-cTnT	high sensitivity cardiac troponin T
HTN	systemic arterial hypertension
ICI	immune checkpoint inhibitors
IVC	inferior vena cava
LV	left ventricular
LVEF	left ventricular ejection fraction
LVD	left ventricular dysfunction
MI	myocardial infarction
MUGA	multigated acquisition
NPs	natriuretic peptides
NT-proBNP	N-terminal pro-B-type natriuretic peptide
PAD	peripheral artery disease
PE	pulmonary embolism
PH	pulmonary hypertension
PI	proteasome inhibitors
ROS	reactive oxygen species
RV	right ventricular
RVEF	right ventricular ejection fraction
TAPSE	tricuspid annular plane systolic excursion
TEE	transesophageal echocardiography
TKIs	small molecule tyrosine kinase inhibitors
TIL	tumor-infiltrating lymphocytes
TR	tricuspid regurgitation
TTE	transthoracic echocardiography
TTS	Takotsubo syndrome
VEGF	vascular endothelial growth factor
VEGFi	vascular endothelial growth factor inhibitors
VHD	valvular heart disease
VTE	venous thrombosis and venous thromboembolism

## References

[1] Loud J., Murphy J. (2017). Cancer Screening and Early Detection in the 21st Century. Semin. Oncol. Nurs..

[2] Zamorano J.L., Lancellotti P., Rodriguez Muñoz D., Aboyans V., Asteggiano R., Galderisi M., Habib G., Lenihan D.J., Lip G.Y.H., Lyon A.R. (2016). 2016 ESC Position Paper on Cancer Treatments and Cardiovascular Toxicity Developed under the Auspices of the ESC Committee for Practice Guidelines: The Task Force for Cancer Treatments and Cardiovascular Toxicity of the European Society of Cardiology (ESC). Eur. Heart J..

[3] Daher I.N., Daigle T.R., Bhatia N., Durand J.-B. (2012). The Prevention of Cardiovascular Disease in Cancer Survivors. Tex. Heart Inst. J..

[4] Plana J.C., Galderisi M., Barac A., Ewer M.S., Ky B., Scherrer-Crosbie M., Ganame J., Sebag I.A., Agler D.A., Badano L.P. (2014). Expert Consensus for Multimodality Imaging Evaluation of Adult Patients during and after Cancer Therapy: A Report from the American Society of Echocardiography and the European Association of Cardiovascular Imaging. Eur. Heart J.—Cardiovasc. Imaging.

[5] Virani S.A., Dent S., Brezden-Masley C., Clarke B., Davis M.K., Jassal D.S., Johnson C., Lemieux J., Paterson I., Sebag I.A. (2016). Canadian Cardiovascular Society Guidelines for Evaluation and Management of Cardiovascular Complications of Cancer Therapy. Can. J. Cardiol..

[6] Curigliano G., Lenihan D., Fradley M., Ganatra S., Barac A., Blaes A., Herrmann J., Porter C., Lyon A.R., Lancellotti P. (2020). Management of Cardiac Disease in Cancer Patients throughout Oncological Treatment: ESMO Consensus Recommendations. Ann. Oncol..

[7] Lyon A.R., López-Fernández T., Couch L.S., Asteggiano R., Aznar M.C., Bergler-Klein J., Boriani G., Cardinale D., Cordoba R., Cosyns B. (2022). 2022 ESC Guidelines on Cardio-Oncology Developed in Collaboration with the European Hematology Association (EHA), the European Society for Therapeutic Radiology and Oncology (ESTRO) and the International Cardio-Oncology Society (IC-OS). Eur. Heart J.

[8] Omerovic E., Citro R., Bossone E., Redfors B., Backs J., Bruns B., Ciccarelli M., Couch L.S., Dawson D., Grassi G. (2022). Pathophysiology of Takotsubo Syndrome—A Joint Scientific Statement from the Heart Failure Association Takotsubo Syndrome Study Group and Myocardial Function Working Group of the European Society of Cardiology—Part 2: Vascular Pathophysiology, Gender and Sex Hormones, Genetics, Chronic Cardiovascular Problems and Clinical Implications. Eur. J. Heart Fail..

[9] Cardinale D., Iacopo F., Cipolla C.M. (2020). Cardiotoxicity of Anthracyclines. Front. Cardiovasc. Med..

[10] Bhatia S. (2020). Genetics of Anthracycline Cardiomyopathy in Cancer Survivors: JACC: CardioOncology State-of-the-Art Review. JACC CardioOncol..

[11] Madeddu C., Deidda M., Piras A., Cadeddu C., Demurtas L., Puzzoni M., Piscopo G., Scartozzi M., Mercuro G. (2016). Pathophysiology of Cardiotoxicity Induced by Nonanthracycline Chemotherapy. J. Cardiovasc. Med..

[12] Marullo R., Werner E., Degtyareva N., Moore B., Altavilla G., Ramalingam S.S., Doetsch P.W. (2013). Cisplatin Induces a Mitochondrial-ROS Response That Contributes to Cytotoxicity Depending on Mitochondrial Redox Status and Bioenergetic Functions. PLoS ONE.

[13] Scott S.S., Greenlee A.N., Matzko A., Stein M., Naughton M.T., Zaramo T.Z., Schwendeman E.J., Mohammad S.J., Diallo M., Revan R. (2022). Intracellular Signaling Pathways Mediating Tyrosine Kinase Inhibitor Cardiotoxicity. Heart Fail. Clin..

[14] Marty M., Cognetti F., Maraninchi D., Snyder R., Mauriac L., Tubiana-Hulin M., Chan S., Grimes D., Antón A., Lluch A. (2005). Randomized Phase II Trial of the Efficacy and Safety of Trastuzumab Combined with Docetaxel in Patients with Human Epidermal Growth Factor Receptor 2-Positive Metastatic Breast Cancer Administered as First-Line Treatment: The M77001 Study Group. J. Clin. Oncol..

[15] Alvi R.M., Frigault M.J., Fradley M.G., Jain M.D., Mahmood S.S., Awadalla M., Lee D.H., Zlotoff D.A., Zhang L., Drobni Z.D. (2019). Cardiovascular Events Among Adults Treated With Chimeric Antigen Receptor T-Cells (CAR-T). J. Am. Coll. Cardiol..

[16] Mitchell J.D., Cehic D.A., Morgia M., Bergom C., Toohey J., Guerrero P.A., Ferencik M., Kikuchi R., Carver J.R., Zaha V.G. (2021). Cardiovascular Manifestations From Therapeutic Radiation: A Multidisciplinary Expert Consensus Statement From the International Cardio-Oncology Society. JACC CardioOncol..

[17] Herrmann J. (2020). Adverse Cardiac Effects of Cancer Therapies: Cardiotoxicity and Arrhythmia. Nat. Rev. Cardiol..

[18] Ederhy S., Cautela J., Ancedy Y., Escudier M., Thuny F., Cohen A. (2018). Takotsubo-Like Syndrome in Cancer Patients Treated With Immune Checkpoint Inhibitors. JACC Cardiovasc. Imaging.

[19] Sinagra G., Anzini M., Pereira N.L., Bussani R., Finocchiaro G., Bartunek J., Merlo M. (2016). Myocarditis in Clinical Practice. Mayo. Clin. Proc..

[20] Zito C., Manganaro R., Carerj S., Antonini-Canterin F., Benedetto F. (2020). Peripheral Artery Disease and Stroke. J. Cardiovasc. Echogr..

[21] Plummer C., Henderson R.D., O’Sullivan J.D., Read S.J. (2011). Ischemic Stroke and Transient Ischemic Attack after Head and Neck Radiotherapy: A Review. Stroke.

[22] De Bruin M.L., Dorresteijn L.D.A., van’t Veer M.B., Krol A.D.G., van der Pal H.J., Kappelle A.C., Boogerd W., Aleman B.M.P., van Leeuwen F.E. (2009). Increased Risk of Stroke and Transient Ischemic Attack in 5-Year Survivors of Hodgkin Lymphoma. J. Natl. Cancer. Inst..

[23] Haddad T.C., Greeno E.W. (2006). Chemotherapy-Induced Thrombosis. Thromb. Res..

[24] Farge D., Frere C., Connors J.M., Khorana A.A., Kakkar A., Ay C., Muñoz A., Brenner B., Prata P.H., Brilhante D. (2022). 2022 International Clinical Practice Guidelines for the Treatment and Prophylaxis of Venous Thromboembolism in Patients with Cancer, Including Patients with COVID-19. Lancet Oncol..

[25] Chiorescu R.M., Mocan M., Stoia M.A., Barta A., Goidescu C.M., Chiorescu S., Farcaş A.D. (2021). Arguments for Using Direct Oral Anticoagulants in Cancer-Related Venous Thromboembolism. Healthcare.

[26] Di Nisio M., Ferrante N., Feragalli B., De Tursi M., Iacobelli S., Cuccurullo F., Porreca E. (2011). Arterial Thrombosis in Ambulatory Cancer Patients Treated with Chemotherapy. Thromb. Res..

[27] Izzedine H., Ederhy S., Goldwasser F., Soria J.C., Milano G., Cohen A., Khayat D., Spano J.P. (2009). Management of Hypertension in Angiogenesis Inhibitor-Treated Patients. Ann. Oncol..

[28] Wu S., Chen J.J., Kudelka A., Lu J., Zhu X. (2008). Incidence and Risk of Hypertension with Sorafenib in Patients with Cancer: A Systematic Review and Meta-Analysis. Lancet Oncol..

[29] Eremina V., Jefferson J.A., Kowalewska J., Hochster H., Haas M., Weisstuch J., Richardson C., Kopp J.B., Kabir M.G., Backx P.H. (2008). VEGF Inhibition and Renal Thrombotic Microangiopathy. N. Engl. J. Med..

[30] Pothineni N.V.K., Van Besien H., Fradley M.G. (2022). Arrhythmic Complications Associated with Cancer Therapies. Heart Fail. Clin..

[31] Farmakis D., Parissis J., Filippatos G. (2014). Insights into Onco-Cardiology: Atrial Fibrillation in Cancer. J. Am. Coll. Cardiol..

[32] Ali A., Hothi S.S., Thompson A., Malik N. (2013). Negative Chronotropic Effects and Coronary Ischaemic Abnormalities Following Thalidomide Therapy. Cardiology.

[33] Chiabrando J.G., Bonaventura A., Vecchié A., Wohlford G.F., Mauro A.G., Jordan J.H., Grizzard J.D., Montecucco F., Berrocal D.H., Brucato A. (2020). Management of Acute and Recurrent Pericarditis: JACC State-of-the-Art Review. J. Am. Coll. Cardiol..

[34] Hu J.-R., Florido R., Lipson E.J., Naidoo J., Ardehali R., Tocchetti C.G., Lyon A.R., Padera R.F., Johnson D.B., Moslehi J. (2019). Cardiovascular Toxicities Associated with Immune Checkpoint Inhibitors. Cardiovasc. Res..

[35] Pedersen L.N., Khoobchandani M., Brenneman R., Mitchell J.D., Bergom C. (2022). Radiation-Induced Cardiac Dysfunction: Optimizing Radiation Delivery and Postradiation Care. Heart Fail. Clin..

[36] McDonagh T.A., Metra M., Adamo M., Gardner R.S., Baumbach A., Böhm M., Burri H., Butler J., Čelutkienė J., Chioncel O. (2021). 2021 ESC Guidelines for the Diagnosis and Treatment of Acute and Chronic Heart Failure. Eur. Heart J..

[37] Habib G., Lancellotti P., Antunes M.J., Bongiorni M.G., Casalta J.-P., Del Zotti F., Dulgheru R., El Khoury G., Erba P.A., Iung B. (2015). 2015 ESC Guidelines for the Management of Infective Endocarditis: The Task Force for the Management of Infective Endocarditis of the European Society of Cardiology (ESC). Endorsed by: European Association for Cardio-Thoracic Surgery (EACTS), the European Association of Nuclear Medicine (EANM). Eur. Heart J..

[38] Vahanian A., Beyersdorf F., Praz F., Milojevic M., Baldus S., Bauersachs J., Capodanno D., Conradi L., De Bonis M., De Paulis R. (2022). 2021 ESC/EACTS Guidelines for the Management of Valvular Heart Disease. Eur. Heart J..

[39] Cutter D.J., Schaapveld M., Darby S.C., Hauptmann M., van Nimwegen F.A., Krol A.D.G., Janus C.P.M., van Leeuwen F.E., Aleman B.M.P. (2015). Risk of Valvular Heart Disease after Treatment for Hodgkin Lymphoma. J. Natl. Cancer Inst..

[40] Heidenreich P.A., Hancock S.L., Lee B.K., Mariscal C.S., Schnittger I. (2003). Asymptomatic Cardiac Disease Following Mediastinal Irradiation. J. Am. Coll. Cardiol..

[41] Hering D., Faber L., Horstkotte D. (2003). Echocardiographic Features of Radiation-Associated Valvular Disease. Am. J. Cardiol..

[42] Humbert M., Kovacs G., Hoeper M.M., Badagliacca R., Berger R.M.F., Brida M., Carlsen J., Coats A.J.S., Escribano-Subias P., Ferrari P. (2022). 2022 ESC/ERS Guidelines for the Diagnosis and Treatment of Pulmonary Hypertension. Eur. Heart J..

[43] Montani D., Bergot E., Günther S., Savale L., Bergeron A., Bourdin A., Bouvaist H., Canuet M., Pison C., Macro M. (2012). Pulmonary Arterial Hypertension in Patients Treated by Dasatinib. Circulation.

[44] Pudil R., Mueller C., Čelutkienė J., Henriksen P.A., Lenihan D., Dent S., Barac A., Stanway S., Moslehi J., Suter T.M. (2020). Role of Serum Biomarkers in Cancer Patients Receiving Cardiotoxic Cancer Therapies: A Position Statement from the Cardio-Oncology Study Group of the Heart Failure Association and the Cardio-Oncology Council of the European Society of Cardiology. Eur. J. Heart Fail..

[45] Kwietniak M., Al-Amawi T., Błaszkowski T., Sulżyc-Bielicka V., Kładny J. (2017). The Usefulness of D-Dimer in Diagnosis and Prediction of Venous Thromboembolism in Patients with Abdominal Malignancy. Pol. Przegl. Chir..

[46] Mueller C., McDonald K., de Boer R.A., Maisel A., Cleland J.G.F., Kozhuharov N., Coats A.J.S., Metra M., Mebazaa A., Ruschitzka F. (2019). Heart Failure Association of the European Society of Cardiology Practical Guidance on the Use of Natriuretic Peptide Concentrations. Eur. J. Heart Fail..

[47] Westermann D., Neumann J.T., Sörensen N.A., Blankenberg S. (2017). High-Sensitivity Assays for Troponin in Patients with Cardiac Disease. Nat. Rev. Cardiol..

[48] Januzzi J.L., Mahler S.A., Christenson R.H., Rymer J., Newby L.K., Body R., Morrow D.A., Jaffe A.S. (2019). Recommendations for Institutions Transitioning to High-Sensitivity Troponin Testing: JACC Scientific Expert Panel. J. Am. Coll. Cardiol..

[49] Finkelman B.S., Putt M., Wang T., Wang L., Narayan H., Domchek S., DeMichele A., Fox K., Matro J., Shah P. (2017). Arginine-Nitric Oxide Metabolites and Cardiac Dysfunction in Patients With Breast Cancer. J. Am. Coll. Cardiol..

[50] Demissei B.G., Freedman G., Feigenberg S.J., Plastaras J.P., Maity A., Smith A.M., McDonald C., Sheline K., Simone C.B., Lin L.L. (2019). Early Changes in Cardiovascular Biomarkers with Contemporary Thoracic Radiation Therapy for Breast Cancer, Lung Cancer, and Lymphoma. Int. J. Radiat. Oncol. Biol. Phys..

[51] Beer L.A., Kossenkov A.V., Liu Q., Luning Prak E., Domchek S., Speicher D.W., Ky B. (2016). Baseline Immunoglobulin E Levels as a Marker of Doxorubicin- and Trastuzumab-Associated Cardiac Dysfunction. Circ. Res..

[52] Rigaud V.O.-C., Ferreira L.R.P., Ayub-Ferreira S.M., Ávila M.S., Brandão S.M.G., Cruz F.D., Santos M.H.H., Cruz C.B.B.V., Alves M.S.L., Issa V.S. (2017). Circulating MiR-1 as a Potential Biomarker of Doxorubicin-Induced Cardiotoxicity in Breast Cancer Patients. Oncotarget.

[53] Suzuki T., Bossone E., Sawaki D., Jánosi R.A., Erbel R., Eagle K., Nagai R. (2013). Biomarkers of Aortic Diseases. Am. Heart J..

[54] Suzuki T., Lyon A., Saggar R., Heaney L.M., Aizawa K., Cittadini A., Mauro C., Citro R., Limongelli G., Ferrara F. (2016). Editor’s Choice-Biomarkers of Acute Cardiovascular and Pulmonary Diseases. Eur. Heart J. Acute Cardiovasc. Care.

[55] Dobric M., Ostojic M., Giga V., Djordjevic-Dikic A., Stepanovic J., Radovanovic N., Beleslin B. (2015). Glycogen Phosphorylase BB in Myocardial Infarction. Clin. Chim. Acta.

[56] Popolo A., Adesso S., Pinto A., Autore G., Marzocco S. (2014). L-Arginine and Its Metabolites in Kidney and Cardiovascular Disease. Amino Acids.

[57] Otaki Y., Watanabe T., Kubota I. (2017). Heart-Type Fatty Acid-Binding Protein in Cardiovascular Disease: A Systemic Review. Clin. Chim. Acta.

[58] Chen Y., Nilsson A.H., Goncalves I., Edsfeldt A., Engström G., Melander O., Orho-Melander M., Rauch U., Tengryd C., Venuraju S.M. (2020). Evidence for a Protective Role of Placental Growth Factor in Cardiovascular Disease. Sci. Transl. Med..

[59] Mauricio R., Singh K., Sanghavi M., Ayers C.R., Rohatgi A., Vongpatanasin W., de Lemos J.A., Khera A. (2022). Soluble Fms-like Tyrosine Kinase-1 (SFlt-1) Is Associated with Subclinical and Clinical Atherosclerotic Cardiovascular Disease: The Dallas Heart Study. Atherosclerosis.

[60] D’Alessandra Y., Devanna P., Limana F., Straino S., Di Carlo A., Brambilla P.G., Rubino M., Carena M.C., Spazzafumo L., De Simone M. (2010). Circulating MicroRNAs Are New and Sensitive Biomarkers of Myocardial Infarction. Eur. Heart J..

[61] Čelutkienė J., Pudil R., López-Fernández T., Grapsa J., Nihoyannopoulos P., Bergler-Klein J., Cohen-Solal A., Farmakis D., Tocchetti C.G., Haehling S. (2020). Role of Cardiovascular Imaging in Cancer Patients Receiving Cardiotoxic Therapies: A Position Statement on Behalf of the H Eart F Ailure A Ssociation (HFA), the E Uropean A Ssociation of C Ardiovascular I Maging (EACVI) and the Cardio-Oncology C Ouncil of the E Uropean S Ociety of C Ardiology (ESC). Eur. J. Heart Fail..

[62] Oikonomou E.K., Kokkinidis D.G., Kampaktsis P.N., Amir E.A., Marwick T.H., Gupta D., Thavendiranathan P. (2019). Assessment of Prognostic Value of Left Ventricular Global Longitudinal Strain for Early Prediction of Chemotherapy-Induced Cardiotoxicity: A Systematic Review and Meta-Analysis. JAMA Cardiol..

[63] Douglas P.S., Khandheria B., Stainback R.F., Weissman N.J., Brindis R.G., Patel M.R., Khandheria B., Alpert J.S., Fitzgerald D., Heidenreich P. (2007). ACCF/ASE/ACEP/ASNC/SCAI/SCCT/SCMR 2007 Appropriateness Criteria for Transthoracic and Transesophageal Echocardiography: A Report of the American College of Cardiology Foundation Quality Strategic Directions Committee Appropriateness Criteria Working Group, American Society of Echocardiography, American College of Emergency Physicians, American Society of Nuclear Cardiology, Society for Cardiovascular Angiography and Interventions, Society of Cardiovascular Computed Tomography, and the Society for Cardiovascular Magnetic Resonance Endorsed by the American College of Chest Physicians and the Society of Critical Care Medicine. J. Am. Coll. Cardiol..

[64] Flachskampf F.A., Decoodt P., Fraser A.G., Daniel W.G., Roelandt J.R., Subgroup on Transesophageal Echocardiography and Valvular Heart Disease, Working Group on Echocardiography of the European Society of Cardiology Guidelines from the Working Group (2001). Recommendations for Performing Transesophageal Echocardiography. Eur. J. Echocardiogr..

[65] Aboyans V., Ricco J.-B., Bartelink M.-L.E.L., Björck M., Brodmann M., Cohnert T., Collet J.-P., Czerny M., De Carlo M., Debus S. (2018). 2017 ESC Guidelines on the Diagnosis and Treatment of Peripheral Arterial Diseases, in Collaboration with the European Society for Vascular Surgery (ESVS): Document Covering Atherosclerotic Disease of Extracranial Carotid and Vertebral, Mesenteric, Renal, Upper and Lower Extremity ArteriesEndorsed by: The European Stroke Organization (ESO)The Task Force for the Diagnosis and Treatment of Peripheral Arterial Diseases of the European Society of Cardiology (ESC) and of the European Society for Vascular Surgery (ESVS). Eur. Heart J..

[66] Isselbacher E.M., Preventza O., Hamilton Black J., Augoustides J.G., Beck A.W., Bolen M.A., Braverman A.C., Bray B.E., Brown-Zimmerman M.M., Chen E.P. (2022). 2022 ACC/AHA Guideline for the Diagnosis and Management of Aortic Disease: A Report of the American Heart Association/American College of Cardiology Joint Committee on Clinical Practice Guidelines. Circulation.

[67] Grothues F., Smith G.C., Moon J.C.C., Bellenger N.G., Collins P., Klein H.U., Pennell D.J. (2002). Comparison of Interstudy Reproducibility of Cardiovascular Magnetic Resonance with Two-Dimensional Echocardiography in Normal Subjects and in Patients with Heart Failure or Left Ventricular Hypertrophy. Am. J. Cardiol..

[68] Contaldi C., Dellegrottaglie S., Mauro C., Ferrara F., Romano L., Marra A.M., Ranieri B., Salzano A., Rega S., Scatteia A. (2021). Role of Cardiac Magnetic Resonance Imaging in Heart Failure. Heart Fail. Clin..

[69] Jordan J.H., Todd R.M., Vasu S., Hundley W.G. (2018). Cardiovascular Magnetic Resonance in the Oncology Patient. JACC Cardiovasc. Imaging.

[70] Thavendiranathan P., Wintersperger B.J., Flamm S.D., Marwick T.H. (2013). Cardiac MRI in the Assessment of Cardiac Injury and Toxicity from Cancer Chemotherapy: A Systematic Review. Circ. Cardiovasc. Imaging.

[71] Galán-Arriola C., Lobo M., Vílchez-Tschischke J.P., López G.J., de Molina-Iracheta A., Pérez-Martínez C., Agüero J., Fernández-Jiménez R., Martín-García A., Oliver E. (2019). Serial Magnetic Resonance Imaging to Identify Early Stages of Anthracycline-Induced Cardiotoxicity. J. Am. Coll. Cardiol..

[72] Bonaca M.P., Olenchock B.A., Salem J.-E., Wiviott S.D., Ederhy S., Cohen A., Stewart G.C., Choueiri T.K., Di Carli M., Allenbach Y. (2019). Myocarditis in the Setting of Cancer Therapeutics: Proposed Case Definitions for Emerging Clinical Syndromes in Cardio-Oncology. Circulation.

[73] Lee S.P., Park J.B., Kim H.K., Kim Y.J., Grogan M., Sohn D.W. (2019). Contemporary Imaging Diagnosis of Cardiac Amyloidosis. J. Cardiovasc. Imaging.

[74] Mousavi N., Cheezum M.K., Aghayev A., Padera R., Vita T., Steigner M., Hulten E., Bittencourt M.S., Dorbala S., Di Carli M.F. (2019). Assessment of Cardiac Masses by Cardiac Magnetic Resonance Imaging: Histological Correlation and Clinical Outcomes. J. Am. Heart Assoc..

[75] Harries I., Liang K., Williams M., Berlot B., Biglino G., Lancellotti P., Plana J.C., Bucciarelli-Ducci C. (2020). Magnetic Resonance Imaging to Detect Cardiovascular Effects of Cancer Therapy: JACC CardioOncology State-of-the-Art Review. Cardio Oncol..

[76] Gambril J.A., Chum A., Goyal A., Ruz P., Mikrut K., Simonetti O., Dholiya H., Patel B., Addison D. (2022). Cardiovascular Imaging in Cardio-Oncology: The Role of Echocardiography and Cardiac MRI in Modern Cardio-Oncology. Heart Fail. Clin..

[77] Soufer A., Liu C., Henry M.L., Baldassarre L.A. (2020). Nuclear Cardiology in the Context of Multimodality Imaging to Detect Cardiac Toxicity from Cancer Therapeutics: Established and Emerging Methods. J. Nucl. Cardiol..

[78] van Nimwegen F.A., Schaapveld M., Janus C.P.M., Krol A.D.G., Petersen E.J., Raemaekers J.M.M., Kok W.E.M., Aleman B.M.P., van Leeuwen F.E. (2015). Cardiovascular Disease after Hodgkin Lymphoma Treatment: 40-Year Disease Risk. JAMA Intern. Med..

[79] Daniëls L.A., Krol A.D.G., de Graaf M.A., Scholte A.J.H.A., Van’t Veer M.B., Putter H., de Roos A., Schalij M.J., Creutzberg C.L. (2014). Screening for Coronary Artery Disease after Mediastinal Irradiation in Hodgkin Lymphoma Survivors: Phase II Study of Indication and Acceptance†. Ann. Oncol..

[80] Meijboom W.B., van Mieghem C.A.G., Mollet N.R., Pugliese F., Weustink A.C., van Pelt N., Cademartiri F., Nieman K., Boersma E., de Jaegere P. (2007). 64-Slice Computed Tomography Coronary Angiography in Patients with High, Intermediate, or Low Pretest Probability of Significant Coronary Artery Disease. J. Am. Coll. Cardiol..

[81] Plana J.C., Thavendiranathan P., Bucciarelli-Ducci C., Lancellotti P. (2018). Multi-Modality Imaging in the Assessment of Cardiovascular Toxicity in the Cancer Patient. JACC Cardiovasc. Imaging.

[82] Knuuti J., Wijns W., Saraste A., Capodanno D., Barbato E., Funck-Brentano C., Prescott E., Storey R.F., Deaton C., Cuisset T. (2020). 2019 ESC Guidelines for the Diagnosis and Management of Chronic Coronary Syndromes. Eur. Heart J..

[83] Ibanez B., James S., Agewall S., Antunes M.J., Bucciarelli-Ducci C., Bueno H., Caforio A.L.P., Crea F., Goudevenos J.A., Halvorsen S. (2018). 2017 ESC Guidelines for the Management of Acute Myocardial Infarction in Patients Presenting with ST-Segment Elevation: The Task Force for the Management of Acute Myocardial Infarction in Patients Presenting with ST-Segment Elevation of the European Society of Cardiology (ESC). Eur. Heart J..

[84] Adler Y., Charron P., Imazio M., Badano L., Barón-Esquivias G., Bogaert J., Brucato A., Gueret P., Klingel K., Lionis C. (2015). 2015 ESC Guidelines for the Diagnosis and Management of Pericardial Diseases: The Task Force for the Diagnosis and Management of Pericardial Diseases of the European Society of Cardiology (ESC)Endorsed by: The European Association for Cardio-Thoracic Surgery (EACTS). Eur. Heart J..

[85] Williams B., Mancia G., Spiering W., Rosei E.A., Azizi M., Burnier M., Clement D.L., Coca A., de Simone G., Dominiczak A. (2019). [2018 ESC/ESH Guidelines for the management of arterial hypertension]. Kardiol. Pol..

[86] Hindricks G., Potpara T., Dagres N., Arbelo E., Bax J.J., Blomström-Lundqvist C., Boriani G., Castella M., Dan G.-A., Dilaveris P.E. (2021). 2020 ESC Guidelines for the Diagnosis and Management of Atrial Fibrillation Developed in Collaboration with the European Association for Cardio-Thoracic Surgery (EACTS). Eur. Heart J..

[87] Collet J.-P., Thiele H., Barbato E., Barthélémy O., Bauersachs J., Bhatt D.L., Dendale P., Dorobantu M., Edvardsen T., Folliguet T. (2021). 2020 ESC Guidelines for the Management of Acute Coronary Syndromes in Patients Presenting without Persistent ST-Segment Elevation. Rev. Esp. Cardiol..

